# Mobile Phone Screen Surfaces as Silent Reservoirs of Multidrug‐Resistant *Staphylococcus aureus* in Healthcare Settings

**DOI:** 10.1155/ijm/3567863

**Published:** 2026-11-08

**Authors:** Mehdi Goudarzi, Arsha Nouzari

**Affiliations:** ^1^ Department of Microbiology School of Medicine Shahid Beheshti University of Medical Sciences Tehran Tehran Province Iran sbmu.ac.ir

## Abstract

**Objectives:**

Mobile phones in healthcare settings function as neglected reservoirs that enable the dissemination of clinically important pathogens.

**Methods:**

This study is aimed at characterizing *Staphylococcus aureus* isolates recovered from healthcare workers′ mobile phone screen surfaces by evaluating antimicrobial resistance profiles, biofilm‐forming capacity, virulence‐associated determinants, and molecular characteristics using multiplex PCR–based coagulase typing methods.

**Results:**

Among 490 individual mobile phone screens collected from healthcare workers, 75 phones (15.3%) yielded confirmed *S. aureus* isolates. All recovered isolates were included for phenotypic and molecular characterization. A substantial proportion of these isolates were identified as methicillin‐resistant *Staphylococcus aureus* (MRSA) (70.7%), and multidrug resistance was observed in 78.7% of the isolates. Notably, all isolates retained full susceptibility to vancomycin and linezolid. Inducible resistance to clindamycin and resistance to mupirocin were detected in 24% and 20% of isolates, respectively. Assessment of biofilm‐forming capacity revealed that 94.7% of the isolates were capable of biofilm production, with strong, moderate, and weak phenotypes observed in 49.3%, 28.2%, and 22.5% of cases, respectively. Molecular characterization demonstrated a high prevalence of key resistance determinants, most prominently *mecA* (66.7%) and *tetM* (52%), followed by genes conferring resistance to aminoglycosides and macrolides, including *ant*(*4*  ^′^)*-Ia* (34.8%), *ermA* (16%), *msrB* (16%), *aac*(*6*  ^′^)*-Ie/aph*(*2*  ^″^) (14.7%), *ermB* (14.7%), *aph*(*3*  ^′^)*-IIIa* (6.7%), and *mecC* (4%). Adhesion and biofilm‐associated genes were dominated by *clfA* (93.3%), *clfB* (90.7%), and *fnbB* (89.3%), followed by *cna* (44%), *fnbA* (42.7%), *ebp* (18.7%), and *bap* (9.3%). Coagulase typing revealed marked genetic heterogeneity, with *coa* Type II (22.7%) being most frequent followed by Types I (18.7%), Vb (16%), III (13.3%), VII (12%), X (10.7%), and IVb (6.6%).

**Conclusions:**

These findings demonstrate that healthcare workers′ mobile phone screen surfaces can harbor multidrug‐resistant and virulence‐associated *S. aureus* isolates. Although contaminated screens may represent potential contact points associated with transmission risk, further studies incorporating longitudinal sampling and molecular epidemiological approaches are required to determine their actual contribution to pathogen transmission.

## 1. Introduction

Mobile phones have become indispensable tools for healthcare workers (HCWs), facilitating rapid communication and immediate access to clinical information during daily medical practice. Throughout routine patient care, these devices are repeatedly handled, frequently exposed to hands, skin, and contaminated hospital environments, and are seldom subjected to systematic disinfection. Such conditions promote microbial survival and accumulation on mobile phone surfaces, positioning these devices as potential intermediaries for indirect pathogen transmission within healthcare settings [1, 2]. Although extensive evidence has demonstrated that hospital‐used mobile phone screens harbor diverse microorganisms, personal electronic devices are rarely incorporated into standard infection prevention and control strategies. This persistent gap increases the likelihood that mobile phone screens contribute to the circulation of healthcare‐associated pathogens, particularly in clinical environments characterized by frequent patient contact and high microbial burden [1–3].

Among the microorganisms recovered from contaminated mobile phone screens, *Staphylococcus aureus* is consistently reported as one of the predominant species. This organism is a common commensal of human skin and nasal mucosa, which facilitates its efficient transfer to frequently touched objects [4]. Beyond colonization, *S. aureus* is a clinically significant opportunistic pathogen capable of causing a wide spectrum of infections, ranging from superficial skin disorders to severe, life‐threatening invasive diseases. Its ability to withstand desiccation and persist on dry, abiotic surfaces further enhances its survival on mobile phone screens and protective cases [5]. The public health relevance of *S. aureus* is further intensified by its exceptional capacity to acquire antimicrobial resistance. In particular, the global dissemination of methicillin‐resistant *Staphylococcus aureus* (MRSA) has substantially limited therapeutic options and continues to impose a major burden on healthcare systems worldwide [2–4]. Through extensive genetic adaptability, *S. aureus* can acquire mobile genetic elements encoding resistance determinants and virulence‐associated factors, suggesting that contaminated mobile phone screens may act as overlooked reservoirs for resistant strains within hospital environments [1, 2, 5].

In addition to antimicrobial resistance, virulence attributes are critical determinants of *S. aureus* persistence and transmission. The expression of surface‐associated proteins, toxins, and, notably, biofilm‐forming capacity enhances bacterial survival under adverse conditions. Biofilm formation is of particular concern, as it confers protection against environmental stress and antimicrobial exposure, whereas mobile phone screens provide favorable abiotic surfaces for sustained biofilm development [1, 2, 6]. Accurate epidemiological assessment of *S. aureus* requires reliable strain differentiation methods. Coagulase typing, based on polymorphic variations within the *coa* gene, offers a practical and cost‐effective approach for evaluating genetic diversity and inferring potential transmission pathways among isolates [7].

Despite documented contamination of mobile phone screens with *S. aureus*, comprehensive data regarding *coa* types, biofilm‐forming potential, and antimicrobial resistance profiles of these isolates remain scarce in many regions [1, 3–5, 7]. Accordingly, the present study is aimed at characterizing *S. aureus* strains isolated from HCWs′ mobile phone screen surfaces by assessing their *coa* types, biofilm production, and phenotypic and genotypic antimicrobial resistance patterns, thereby providing information regarding the molecular and phenotypic characteristics of mobile phone‐associated *S. aureus* isolates in healthcare settings.

## 2. Materials and Methods

### 2.1. Sampling and Identification of *S. Aureus*

From August 2024 to July 2025, a total of 490 individual mobile phone screens belonging to HCWs employed in hospitals affiliated with Shahid Beheshti University of Medical Sciences, Tehran, Iran, were investigated. Each mobile phone represented one independent sampling unit, and one sterile swab sample was collected from each phone screen. Therefore, 490 mobile phone samples were prepared and processed. Sample collection was performed using sterile cotton swabs moistened with normal saline, which were gently rubbed over the surfaces of mobile phone screens to ensure adequate microbial recovery. The swabs were immediately inoculated into Trypticase Soy Broth (TSB) (Merck, Germany) and incubated at 37°C for 18–24 h to allow bacterial enrichment. Following incubation, enriched cultures were streaked onto Mannitol Salt Agar plates and incubated overnight under aerobic conditions to facilitate the selective isolation of presumptive *S. aureus* colonies [5, 8]. Colonies showing characteristic morphology, including yellow colonies associated with mannitol fermentation, were selected for further identification. Presumptive isolates were evaluated using conventional microbiological and biochemical tests, including Gram staining, catalase testing, and coagulase testing. Isolates exhibiting phenotypic characteristics consistent with *S. aureus* were subsequently subjected to molecular confirmation by polymerase chain reaction (PCR) targeting the species‐specific *nucA* gene. The combination of phenotypic identification and *nucA*‐based molecular confirmation was used for final identification of *S. aureus* isolates included in this study [8]. For each positive mobile phone sample, a single representative *S. aureus* colony was selected for further characterization. Therefore, only one isolate per positive phone was included in subsequent antimicrobial susceptibility testing, biofilm assays, and molecular analyses. Among the 490 screened mobile phone screens, 75 samples yielded PCR‐confirmed *S. aureus* isolates, corresponding to a contamination rate of 15.3%. A total of 75 *S. aureus* isolates were recovered and included in subsequent antimicrobial susceptibility testing, biofilm assessment, and molecular characterization analyses.

### 2.2. Antimicrobial Susceptibility Testing

Antimicrobial susceptibility testing of confirmed *S. aureus* isolates was performed using the Kirby–Bauer disk diffusion method on Mueller–Hinton agar (MHA) according to the Clinical and Laboratory Standards Institute (CLSI) guidelines (2024). Bacterial suspensions were prepared from overnight cultures and adjusted to a 0.5 McFarland turbidity standard before inoculation. The standardized bacterial suspension was evenly spread onto MHA plates, and antimicrobial disks were subsequently applied. The tested antimicrobial agents included penicillin (PEN, 10 units), nitrofurantoin (NIT, 300 *μ*g), clindamycin (CLI, 2 *μ*g), rifampin (RIF, 5 *μ*g), linezolid (LIN, 30 *μ*g), quinupristin–dalfopristin (SYN, 15 *μ*g), erythromycin (ERY, 15 *μ*g), ciprofloxacin (CIP, 5 *μ*g), gentamicin (GEN, 10 *μ*g), trimethoprim–sulfamethoxazole (SXT, 1.25/23.75 *μ*g), and tetracycline (TET, 30 *μ*g). Isolates were incubated at 35°C±2°C for 16–18 h under aerobic conditions, and inhibition zone diameters were measured in millimeters. Results were interpreted according to CLSI 2024 breakpoint criteria. Phenotypic detection of methicillin resistance was performed using cefoxitin disk diffusion according to CLSI 2024 guidelines. Cefoxitin susceptibility testing was used as a surrogate marker for *mecA*‐mediated methicillin resistance, and molecular among MRSA isolates confirmation was performed by PCR detection of *mecA* and *mecC* resistance determinants. Isolates exhibiting resistance to three or more distinct antimicrobial classes were classified as multidrug resistant (MDR). To ensure methodological accuracy and reproducibility, reference strains *S. aureus* ATCC 25923, ATCC 43300, and ATCC 29213 were included as quality control organisms throughout susceptibility testing procedures. MIC determination for vancomycin and mupirocin was performed using the broth microdilution method according to CLSI 2024 recommendations. Twofold serial dilutions of each antimicrobial agent were prepared, and bacterial inoculum was standardized to the recommended concentration. MIC values were defined as the lowest antimicrobial concentration that completely inhibited visible bacterial growth after incubation. The macrolide–lincosamide–streptogramin B (MLS_B_) resistance phenotypes were determined using the double‐disk diffusion method (D‐test) according to CLSI 2024 recommendations. Briefly, ERY (15 *μ*g) and CLI (2 *μ*g) disks were placed on MHA inoculated with a standardized bacterial suspension adjusted to 0.5 McFarland. The distance between the edges of the ERY and CLI disks was maintained at 15 mm. After incubation at 35°C±2°C for 16–18 h, inhibition zones were interpreted according to CLSI criteria. Flattening of the CLI inhibition zone adjacent to the ERY disk was interpreted as inducible CLI resistance (iMLS_B_), resistance to both ERY and CLI was classified as constitutive MLS_B_ resistance (cMLS_B_), and ERY resistance with CLI susceptibility without zone distortion was classified as the MS phenotype.

### 2.3. Biofilm Formation Assay

The ability of *S. aureus* isolates to produce biofilm was determined using the microtiter plate (MtP) method with minor modifications from previously described protocols [8]. Briefly, bacterial colonies obtained from overnight cultures were suspended and adjusted to a turbidity equivalent to 0.5 McFarland standard (approximately 1.5 × 10^8^ CFU/mL). The adjusted suspensions were subsequently diluted in TSB containing 1% glucose, and 200 *μ*L of each bacterial preparation was added to individual wells of sterile 96‐well flat‐bottom MtPs. The plates were incubated statically at 37°C for 24 h to facilitate biofilm development.

Following incubation, the culture medium containing nonadherent cells was carefully discarded, and each well was washed three times with sterile phosphate‐buffered saline (PBS). The remaining attached bacterial biomass was fixed and stained with 0.1% crystal violet. After removal of the unbound dye, the stained biofilm was solubilized and the absorbance was measured at 490 nm using a microplate reader. All experiments were conducted in triplicate, and wells containing only sterile culture medium were included as negative controls.

For quantitative interpretation, the biofilm cutoff value (ODc) was determined based on the mean optical density of negative control wells plus three standard deviations. According to the obtained OD values, isolates were categorized as nonbiofilm producers, weak, moderate, or strong biofilm producers based on the established classification criteria described previously [8].

### 2.4. Detection of Resistance and Adhesion‐Related Genes

Genomic DNA was extracted from confirmed *S. aureus* isolates using a phenol–chloroform extraction method [8]. PCR assays with related primer sequences were performed to characterize resistance and adhesion‐associated determinants (Table 1). Among phenotypically confirmed MRSA isolates, the methicillin resistance genes *mecA* and *mecC* were detected using gene‐specific primers and previously described PCR conditions. Amplified PCR products were visualized by agarose gel electrophoresis. Additional resistance‐associated genes, including macrolide (*ermA*, *ermB*, *ermC*, *msrA*, and *msrB*), TET (*tetM*), aminoglycoside (*ant*(*4*  ^′^)*-Ia*, *aac*(*6*  ^′^)*-Ie/aph*(*2*  ^″^), *aph*(*3*  ^′^)*-IIIa*), glycopeptide (*vanA* and *vanB*), and mupirocin resistance determinants (*mupA* and *mupB*), as well as adhesion‐associated genes (*fnbA*, *fnbB*, *clfA*, *clfB*, *bap*, *cna*, and *ebp*), were evaluated using the same PCR‐based approach [8].

**Table 1:** Primer sequences, target genes, and references used for detection of antimicrobial resistance and adhesion‐associated genes in *S. aureus* isolates.

Target	Primer	Primer sequence (5 ^′^ → 3 ^′^)	Product size (bp)	Reference
*femA*	F	CTTACTTACTGCTGTACCTG	648	[8]
	R	ATCTCGCTTGTTGTGTGC		
*nucA*	F	GCGATTGATGGTGATACGGTT	270	[8]
	R	AGCCAAGCCTTGACGAACTAAAGC		
*mecA*	F	AGAAGATGGTATGTGGAAGTTAG	583	[8]
	R	ATGTATGTGCGATTGTATTGC		
*luk-PV*	F	TTCACTATTTGTAAAAGTGTCAGACCCACT	180	[8]
	R	TACTAATGAATTTTTTTATCGTAAGCCCTT		
*tst-1*	F	TTATCGTAAGCCCTTTGTTG	398	[8]
	R	TAAAGGTAGTTCTATTGGAGTAGG		
*eta*	F	GCAGGTGTTGATTTAGCATT	93	[8]
	R	AGATGTCCCTATTTTTGCTG		
*etb*	F	ACAAGCAAAAGAATACAGCG	226	[8]
	R	GTTTTTGGCTGCTTCTCTTG		
*ant*(*4* ^′^)*-Ia*	F	AATCGGTAGAAGCCCAA	135	[8]
	R	GCACCTGCCATTGCTA		
*aac*(*6* ^′^)*-Ie/aph*(*2* ^″^)	F	CCAAGAGCAATAAGGGCATACC	222	[8]
	R	CACACTATCATAACCACT		
*aph*(*3* ^′^)*-IIIa*	F	CTTGATCGAAAAATACCGCTGC	269	[8]
	R	TCATACTCTTCCGAGCAAA		
*ermA*	F	TATCTTATCGTTGAGAAGGGATT	139	[8]
	R	CTACACTTGGCTGATGAAA		
*ermB*	F	CTATCTGATTGTTGAAGAAGCATT	141	[8]
	R	GTTTACTCTTGGTTTAGGATCAAA		
*ermC*	F	AATCGTCAATTCCTGCATGT	299	[8]
	R	TAATCGTGGAATACGGGTTTG		
*msrA*	F	GGCACAATAAGAGTG TTTAAAGG	940	[8]
	R	AAGTTATATCATGAATAGATTGTCCTGTT		
*msrB*	F	TATGATATCCATAATAATTATCCAATC	595	[8]
	R	AAGTTATATCATGAATAGATTGTCCTGTT		
*mupA*	F	CCCATGGCTTACCAGTTGA	1158	[8]
	R	CCATGGAGCACTATCCGA		
*tetM*	F	AGTGGAGCGATTACAGAA	158	[8]
	R	CATATGTCCTGGCGTGTCTA		

### 2.5. Staphylococcal Cassette Chromosome *mec* (SCC*mec*) Typing

Molecular characterization of staphylococcal cassette chromosome *mec* (SCC*mec*) elements was carried out using a multiplex PCR‐based approach [8]. To validate assay performance and ensure accurate SCC*mec* assignment, reference strains representing SCC*mec* Types I through VI were included as positive controls. These consisted of Type I (*S. aureus* ATCC 10442), Type II (*S. aureus* N315), Type III (*S. aureus* 85/2082), Type IV (*S. aureus* MW2), Type V (*S. aureus* WIS 173), and Type VI (*S. aureus* HDE288).

### 2.6. *Coa* Typing

*Coa* typing of *S. aureus* isolates was performed using a multiplex PCR protocol based on coagulase gene polymorphisms (Table 2). To enable classification into *coa* Types I to X, four independent PCR reaction sets were applied. In the first multiplex assay, primer combinations were used to simultaneously detect *coa* Types I, II, III, IVa, IVb, Va, and VI. A second multiplex reaction targeted *coa* Types VII, VIII, and X, whereas a third reaction set was designed to identify *coa* Types IX and Vb [7]. Due to sequence similarity between certain coagulase variants, a separate type‐specific PCR assay was employed to distinguish between *coa* Types IVa and IVb. This structured PCR strategy allowed reliable assignment of *S. aureus* isolates to their respective *coa* types.

**Table 2:** Primer sequences for *coa* typing.

Gene	Primer	type *Coa*	Sequence	PCR product	Reference
*Sc-R1*	Coa‐ant1	Common	GGGCAATTACATTTTGGAGGA	—	[7]
	Coa7	Common	TGTTCCATCGTTGTATTCACG	—	[7]
	cot1	I	ATTTTTTGTATTCCTCATACTGCA	bp 368	[7]
	cot2	II	CTTTCGCTTCTTTATAGATAGGATC	bp 288	[7]
	cot3	III	TCAAGTCTGAATTCTTATCC	bp 549	[7]
	cot4	Iva, IVb	AGCATTATGACCATATTGGC	bp 665	[7]
	cot5	Va	TTACCTTGAGTCCCAATTTG	bp 1105	[7]
	cot6	VI	CTATAATCATGCTTATCCCA	bp 850	[7]
*Sc-R2*	cot7	VII	TCAAATCAATTTTCGCCCTA	bp 693	[7]
	cot8	VIII	GATTTTTTATTACTCCCCAGTAATA	bp 210	[7]
	cot10	X	ACTTAATATCCTTGTCATTAGTTG	bp 314	[7]
*Sc-R3*	cot9	IX	ATATACCGTTAGTTACACGC	bp 591	[7]
	Cot1	Vb	AATCATAAAATTTCACCGGGC	bp 411	[7]
*Sc-R4*	F4‐8	type*Coa*	TTACAGTTGGTACAACTGAAGAAGC	bp 455	[7]
	F8‐12	Common	GCCAAAATACCCAACGATGGAACAG	bp 415	[7]

### 2.7. Statistical Analysis

The association between biofilm production categories and MDR phenotypes was evaluated using the chi‐square test. Differences in gene prevalence between MRSA and methicillin‐susceptible *Staphylococcus aureus* (MSSA) groups were evaluated using chi‐square or Fisher′s exact test where appropriate. Odds ratios with 95% confidence intervals were calculated, and *p* values < 0.05 were considered statistically significant. Due to the exploratory nature of the molecular characterization analyses, results were interpreted cautiously and descriptive findings were distinguished from statistically tested associations.

## 3. Results

### 3.1. Prevalence of *S. aureus* and Antimicrobial Resistance Profile

Analysis of 490 individual mobile phone screen samples collected from HCWs revealed that 75 phones were contaminated with *S. aureus*. Among the 75 positive mobile phone screen samples, one representative *S. aureus* isolate from each positive phone was analyzed. Therefore, all reported phenotypic and genotypic characteristics represent 75 independent *S. aureus* isolates obtained from 75 individual contaminated phone screens. Among the confirmed isolates, 53 strains (70.7%) exhibited phenotypic characteristics consistent with MRSA, whereas the remaining 22 isolates (29.3%) were categorized as MSSA. Antimicrobial susceptibility profiling demonstrated marked heterogeneity in resistance patterns among the isolates. The highest level of resistance was observed against PEN, with 81.3% of isolates classified as resistant. In contrast, resistance to NIT was comparatively infrequent, detected in only 12% of the isolates.

A comprehensive overview of the antimicrobial susceptibility profiles of *S. aureus* isolates recovered from mobile phone screens is presented in Table 3. Evaluation of resistance across antimicrobial classes indicated a high prevalence of MDR. Specifically, 59 isolates (78.7%) met the criteria for MDR phenotypes. MDR isolates were identified among both MRSA and MSSA groups. Although MDR was more frequently observed among MRSA isolates, a proportion of MSSA isolates (27.3%; 6/22) also fulfilled the criteria for MDR, indicating that MDR determinants were not exclusively associated with methicillin‐resistant strains. Further analysis revealed considerable diversity within MDR profiles, with a total of 12 distinct resistance patterns identified among the isolates.

**Table 3:** Antimicrobial susceptibility pattern of *S. aureus* strains obtained from mobile phone screens.

Antibiotic^a^	53 MRSA *n* (%)	22 MSSA *n* (%)	Total *n* (%)
PEN	49 (80.3)	12 (19.7)	61 (81.3)
GEN	33 (71.7)	13 (28.3)	46 (61.3)
TET	43 (75.4)	14 (24.6)	57 (76)
ERY	28 (73.7)	10 (26.3)	38 (50.7)
CIP	23 (62.2)	14 (37.8)	37 (49.3)
CLI	16 (80)	4 (20)	20 (26.7)
RIF	18 (85.7)	3 (14.3)	21 (28)
SYN	8 (80)	2 (20)	10 (13.3)
NIT	9 (100)	0 (0)	9 (12)
MUP	15 (100)	0 (0)	15 (20)
SXT	12 (100)	0 (0)	12 (16)

^a^CIP, ciprofloxacin; CLI, clindamycin; ERY, erythromycin; GEN, gentamicin; MUP, mupirocin; NIT, nitrofurantoin; PEN, penicillin; RIF, rifampicin; SXT, trimethoprim–sulfamethoxazole; SYN, quinupristin–dalfopristin; TET, tetracycline.

Broth microdilution analysis demonstrated that all *S. aureus* isolates were susceptible to vancomycin, whereas 20% of tested isolates were resistance to mupirocin according to CLSI 2024 criteria. MIC distribution of vancomycin and mupirocin among *S. aureus* isolates is presented in Table 4.

**Table 4:** MIC distribution of vancomycin and mupirocin among *S. aureus* isolates.

Antibiotic	MIC range (*μ*g/mL)	MIC50	MIC90
Vancomycin	0.5–2	1	2
Mupirocin	0.25–256	0.5	256

Evaluation of MDR profiles revealed considerable heterogeneity among the *S. aureus* isolates. Several resistance patterns were repeatedly identified, with the most prevalent combinations including PEN–GEN–TET–ERY (10.7%; 8/75), followed by PEN–GEN–TET–ERY–CLI–CIP, which accounted for 9.3% of isolates (7/75). Additional frequently detected profiles comprised PEN–ERY–CLI–CIP–RIF (9.3%; 7/75), PEN–GEN–TET–RIF–MUP (8%; 6/75), and PEN–GEN–TET–ERY–CLI (8%; 6/75). These findings highlight the broad spectrum of antimicrobial classes involved in resistance among the recovered isolates.

Phenotypic characterization of CLI resistance demonstrated the presence of MLS_B_ resistance mechanisms. Constitutive MLS_B_ resistance (cMLS_B_ phenotype) was identified in 20 isolates, representing 26.7% of the total collection. In addition, inducible CLI resistance (iMLS_B_ phenotype) was detected in 18 isolates (24%), indicating a substantial proportion of strains with inducible resistance potential. No isolate exhibiting the MS phenotype was detected in this study. Assessment of mupirocin susceptibility showed that 15 isolates (20%) were resistant to this agent. Among mupirocin‐resistant isolates, high‐level mupirocin resistance was predominant, with 10 strains (66.7%) exhibiting a high‐level resistance phenotype (HLMUPR).

### 3.2. Biofilm Formation

Quantitative evaluation of biofilm‐forming capacity using the MtP assay demonstrated that the vast majority of *S. aureus* isolates were capable of biofilm formation. Biofilm production was detected in 71 of the 75 isolates (94.7%), whereas four isolates (5.3%) were classified as nonbiofilm producers. Among all analyzed isolates, 35 (46.7%) were strong biofilm producers, 20 (26.7%) showed moderate biofilm production, and 16 (21.3%) were weak biofilm producers. Of note, all nonbiofilm‐producing strains were identified as methicillin‐MSSA, indicating an absence of these strains among methicillin‐resistant isolates. The distribution of antimicrobial resistance patterns varied across different biofilm production categories. Biofilm‐producing and nonbiofilm‐producing isolates showed distinct resistance profiles; however, statistical association between biofilm intensity and antimicrobial resistance was not assessed. A significant association was observed between biofilm production category (*n* = 71) and MDR phenotype (*n* = 59) among *S. aureus* isolates (*χ* ^2^ = 32.10, *p* < 0.001). MDR was detected in 100% (35/35) of strong biofilm producers, 80% (16/20) of moderate producers, 50% (8/16) of weak producers, and 0% (0/4) of nonbiofilm producers. A detailed overview of antimicrobial resistance patterns observed among biofilm‐producing *S. aureus* isolates is provided in Table 5.

**Table 5:** Distribution of antimicrobial resistance patterns according to biofilm production status among *S. aureus* isolates (*n* = 75).

Biofilm status	Phenotypic resistance^a^ (no; %)	Number^b^ (%)
Strong producer	PEN, GEN, TET, ERY, SYN, NIT, SXT (3; 8.6)	35 (46.7)
	PEN, GEN, TET, ERY, CIP, SYN (5; 14.3)	
	PEN, GEN, TET, ERY, CLI, CIP (7; 20)	
	PEN, GEN, TET, SXT, MUP (5; 14.3)	
	PEN, GEN, TET, RIF, MUP (6; 17.1)	
	PEN, TET, MUP, SXT, RIF (4; 11.4)	
	PEN, GEN, TER, ERY, CLI (3; 8.6)	
	PEN, GEN, TET, ERY (2; 5.7)	
Moderate producer	PEN, GEN, TER, ERY, CLI (3; 15)	20 (26.7)
	PEN, ERY, CLI, CIP, RIF (3; 15)	
	PEN, GEN, TET, ERY (6; 30)	
	PEN, TET, CIP, NIT (2; 10)	
	PEN, GEN, ERY (2; 10)	
	TET, CIP (4; 20)	
Weak producer	PEN, ERY, CLI, CIP, RIF (4; 24.9)	16 (21.3)
	PEN, CIP, SYN, NIT (2; 12.5)	
	PEN, TET, CIP, NIT (2; 12.5)	
	GEN, RIF (3; 18.8)	
	TET, CIP (3; 18.8)	
	TET, CIP (2; 12.5)	
Non producer	PEN, CIP (2; 50)	4 (5.3)
	GEN, RIF (1; 25)	
	CIP (1; 25)	

^a^CIP, ciprofloxacin; CLI, clindamycin; ERY, erythromycin; GEN, gentamicin; MUP, mupirocin; NIT, nitrofurantoin; PEN, penicillin; RIF, rifampicin; SXT, trimethoprim–sulfamethoxazole; SYN, quinupristin–dalfopristin; TET, tetracycline.

^b^Values in the biofilm category column are presented as number (%) based on the total number of *S. aureus* isolates (*n* = 75). Resistance pattern percentages within each biofilm category were calculated using the corresponding subgroup size as the denominator.

### 3.3. Resistance, Toxin, and Biofilm Genes Detection

The distribution of antimicrobial resistance determinants was investigated among all 75 *S. aureus* isolates. Analysis of macrolide resistance‐associated genes indicated that *erm*(*A*) was the most frequently detected determinant, present in 16% of isolates (12/75). This was followed by *erm*(*B*), identified in 14.7% of isolates (11/75), whereas *erm*(*C*) was detected at a considerably lower frequency (2.7%; 2/75) (Figure 1). Further stratification based on CLI resistance phenotypes revealed distinct genetic profiles. Among isolates exhibiting the cMLS_B_ phenotype (*n* = 20), the prevalence of *erm*(*A*) and *msrB* was highest, each detected in 30% of isolates. In contrast, *erm*(*B*) and *erm*(*C*) were each identified in 10% of cMLS_B_ isolates, whereas *msrA* was detected in 5%. In isolates expressing the iMLS_B_ phenotype, *erm*(*B*) emerged as the dominant macrolide resistance gene, with a detection rate of 38.9%. Screening for TET resistance determinants demonstrated that *tet*(*M*) was the most widespread resistance gene overall, identified in 52% of the isolates (39/75). The *tet*(*M*) gene was detected in 30/53 (56.6%) MRSA isolates and 9/22 (40.9%) MSSA isolates. Although *tet*(*M*) carriage was more frequently observed among MRSA isolates, this difference was not statistically significant (*χ* ^2^ = 1.53, *p* = 0.215). PCR‐based detection of aminoglycoside‐modifying enzyme genes revealed relatively limited distribution across the isolate collection. *Ant*(*4*  ^′^)*-Ia* was detected in 16/75 (21.3%) isolates, followed by *aac*(*6*  ^′^)*-Ie/aph*(*2*  ^″^) in 11/75 (14.7%) and *aph*(*3*  ^′^)*-IIIa* in 5/75 (6.7%) isolates. Detection of methicillin resistance‐associated genes.

**Figure 1:**
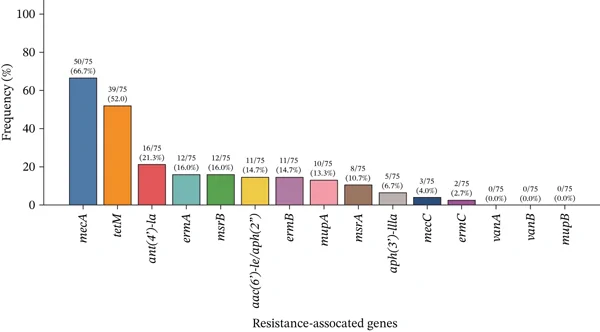
Frequency distribution of antimicrobial resistance‐associated genes in *S. aureus* isolates. This bar chart shows the frequency of antimicrobial resistance‐associated genes detected among the 75 *S. aureus* isolates. Values on the bars are expressed as *n*/*N* (%), where *N* = 75. Genes with no positive isolates in the current collection (*vanA*, *vanB*, and *mupB*) are shown as 0% to provide a complete overview of all investigated resistance determinants.

Among the 53 phenotypically confirmed MRSA isolates, PCR analysis was performed to determine the presence of methicillin resistance‐associated determinants. The *mecA* gene was detected in 35/53 (66.0%) of MRSA isolates, whereas the *mecC* gene was identified in 2/53 (3.8%) isolates. Both *mecC*‐positive isolates were subsequently included in the molecular characterization analysis.

Evaluation of toxin gene carriage among the examined *S. aureus* isolates revealed that 22 of the 75 strains (29.3%) harbored at least one toxin‐encoding gene and were therefore classified as toxigenic. Among the toxin determinants investigated, the *pvl* gene was the most prevalent, detected in 20% of isolates. This was followed by the toxic *tst*, which was identified in 6.7% of strains, whereas the *eta* gene was detected at a lower frequency (2.6%) (Figure 2).

**Figure 2:**
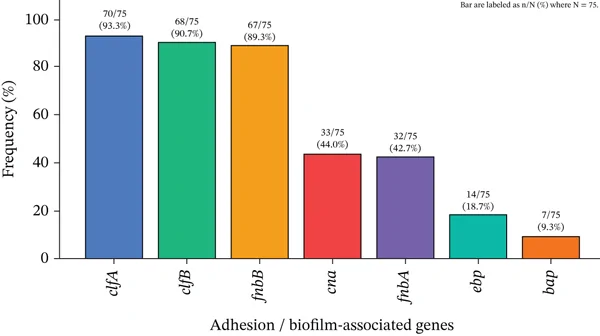
Frequency distribution of adhesion and biofilm‐associated genes in *S. aureus* isolates. This bar chart illustrates the frequency of adhesion and biofilm‐associated genes among the 75 *S. aureus* isolates. Values on the bars are shown as *n*/*N* (%), where *N* = 75.

Molecular screening of genes associated with biofilm formation and bacterial adhesion demonstrated a high overall burden of virulence‐related determinants within the isolate collection (Figure 2). Among the adhesion‐associated genes, *clfA* was detected in 70/75 (93.3%) isolates, followed by *clfB* in 68/75 (90.7%) and *fnbB* in 67/75 (89.3%) isolates. Other detected genes included *cna* (33/75, 44%), *fnbA* (32/75, 42.7%), *ebp* (14/75, 18.7%), and *bap* (7/75, 9.3%).

Combinatorial genotypic analysis of biofilm‐ and adhesion‐related genes revealed substantial diversity, with 10 distinct gene profiles identified among the isolates. The most commonly observed gene combinations were *clfA–clfB–fnbA–fnbB* (21.3%; 16/75), *clfA–clfB–fnbA–fnbB–cna–ebp* (18.7%; 14/75), and *clfA–clfB–fnbA–fnbB–cna* (18.7%; 14/75). Additional prevalent patterns included *clfA–clfB–fnbA* (10.7%; 8/75), *clfA–fnbA–fnbB* (6.7%; 5/75), and *clfA–clfB–fnbA–fnbB–cna–bap* (6.7%; 5/75). Collectively, these findings indicate marked heterogeneity in virulence gene composition among *S. aureus* isolates recovered from HCWs′ mobile phone screens. Percentage distribution of adhesion‐associated genes among biofilm‐producing and nonbiofilm‐producing *S. aureus* isolates are presented in Figure 3.

**Figure 3:**
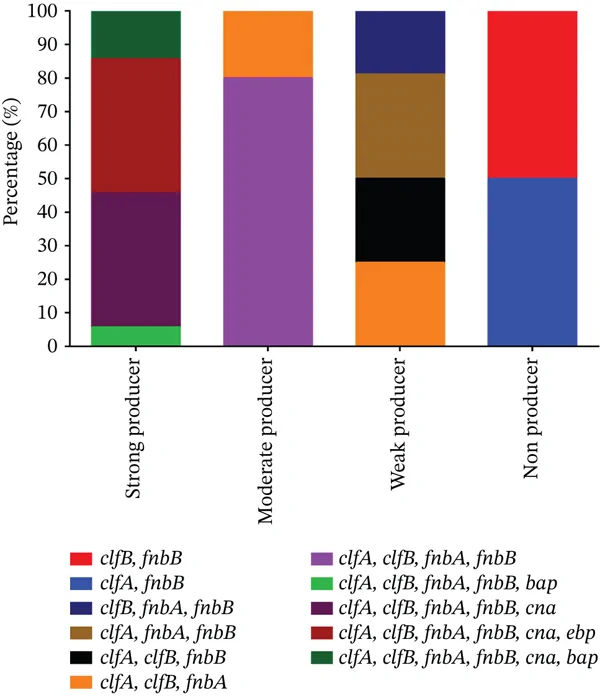
Distribution of adhesion‐associated genes among biofilm‐producing and nonbiofilm‐producing *S. aureus* isolates.

### 3.4. SCC*mec* Typing

SCC*mec* typing of the 53 MRSA isolates revealed the presence of four distinct cassette types, namely SCC*mec* II, III, IV, and V. Among these, SCC*mec* type IV was the most frequently identified, accounting for nearly half of the isolates (47.1%; 25/53). SCC*mec* Types II and III were detected at comparable frequencies, each representing 18.9% of isolates (10/53), whereas SCC*mec* type V was observed in a smaller proportion of strains (15.1%; 8/53). No isolates carrying SCC*mec* Types I or VI were detected, and all MRSA isolates generated interpretable SCC*mec* PCR profiles. Analysis stratified by biofilm‐forming capacity demonstrated a strong association between SCC*mec* type and biofilm intensity among MRSA isolates. Within the group of strong biofilm‐producing MRSA strains, SCC*mec* Type IV was overwhelmingly predominant, detected in 74.1% of isolates (20/27). The remaining strong biofilm‐forming MRSA isolates carried SCC*mec* Type III (14.8%; 4/27), Type II (7.4%; 2/27), or Type V (3.7%; 1/27).

Among MRSA isolates exhibiting moderate biofilm‐forming ability, SCC*mec* Types II and IV were equally prevalent, each identified in 28.6% of isolates (four isolates each). In contrast, SCC*mec* Types III and V were less frequently detected within this group, accounting for 21.4% of isolates each (three isolates per type). In addition to MRSA, a subset of MSSA isolates also demonstrated strong biofilm‐forming capacity. Specifically, eight MSSA isolates were classified as strong biofilm producers, representing 36.4% (8/22) of the MSSA population.

### 3.5. *Coa* Typing

Molecular characterization based on coagulase gene polymorphism revealed pronounced heterogeneity among the 75 *S. aureus* isolates, with seven distinct *coa* types identified, namely, II, I, Vb, III, VII, X, and IVb. The distribution of these types varied considerably, with *coa* type II representing the largest proportion of isolates (22.7%; 17/75), followed by Types I (18.7%; 14/75), Vb (16%; 12/75), III (13.3%; 10/75), VII (12%; 9/75), X (10.7%; 8/75), and IVb (6.6%; 5/75). These findings demonstrate diversity among *coa* types and the investigated molecular markers within the analyzed *S. aureus* isolates. Analysis of the isolates demonstrated distinct *coa* type distributions between MSSA and MRSA isolates. Among MSSA strains, four *coa* types were identified, with type X predominating (36.4%), followed by Types III (27.3%), IVb (22.7%), and VII (13.6%). In contrast, MRSA isolates were predominantly associated with *coa* Types II (32.1%), I (26.4%), and Vb (22.6%), whereas lower frequencies were observed for Types VII (11.3%) and III (7.6%). Notably, approximately one‐third of MSSA isolates assigned to *coa* Type VII and nearly two‐fifths of isolates belonging to *coa* type IVb lacked detectable biofilm‐forming ability. In comparison, isolates representing other *coa* types exhibited variable levels of biofilm production.

Correlation analysis between *coa* types, SCC*mec* elements, and mupirocin resistance demonstrated that mupirocin‐resistant isolates were most frequently associated with the *coa* type II/SCC*mec* III genotype (46.6%; 7/15). Additional associations included *coa* Type II/SCC*mec* IV and *coa* Type I/SCC*mec* IV, each accounting for 26.7% of mupirocin‐resistant isolates (4/15). Among isolates exhibiting high‐level mupirocin resistance and harboring the *mupA* gene, the majority were classified as *coa* Type II/SCC*mec* III (70%; 7/10), whereas the remaining isolates belonged to *coa* Type II/SCC*mec* IV (30%; 3/10). Distribution of MLS_B_ resistance phenotypes further highlighted the diversity among *coa* types. Isolates expressing the iMLS_B_ phenotype were mainly distributed among *coa* Type I/SCC*mec* IV (38.9%; 7/18) and *coa* Type X (33.3%; 6/18), with smaller proportions associated with *coa* Type II/SCC*mec* III (16.7%; 3/18) and *coa* type I/SCC*mec* II (11.1%; 2/18). In contrast, constitutive cMLS_B_ was predominantly observed in isolates classified as *coa* type Vb/SCC*mec* V (38.9%; 8/20) and *coa* type Vb/SCC*mec* IV (38.9%; 4/20). Additionally, cMLS_B_ expression was detected in two‐thirds of *coa* Type II MSSA isolates, as well as in 25% of *coa* Type III/SCC*mec* II isolates and 17.6% of *coa* Type II/SCC*mec* III isolates. Comprehensive genotypic analysis of adhesion‐related determinants identified 11 distinct gene combination patterns among the isolates, with their distribution across *coa* types illustrated in Figure 4.

**Figure 4:**
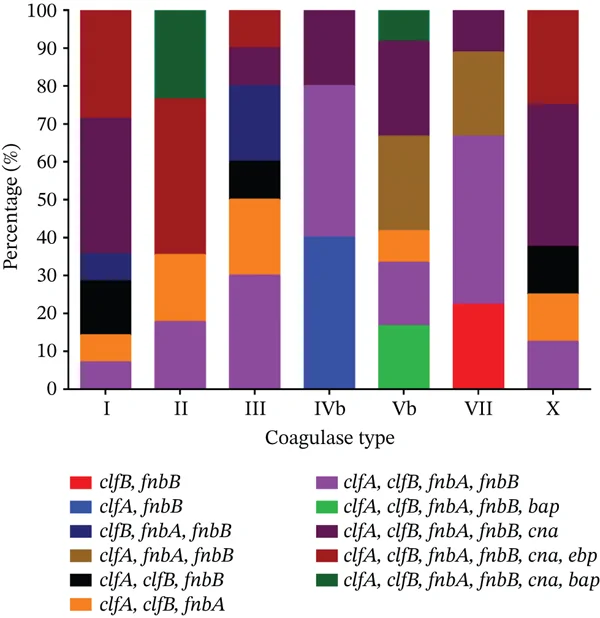
Distribution of adhesion‐associated genes among different *coa* types of *S. aureus.*

Furthermore, all three isolates carrying the *mecC* gene were assigned to *coa* type I/SCC*mec* IV. Collectively, these findings indicate diversity in *coa* types and associated molecular markers among the investigated *S. aureus* isolates. A detailed summary of *coa* type distribution and associated phenotypic and genotypic characteristics is provided in Table 6.

**Table 6:** Characteristics of the 75 *S. aureus* isolates recovered from 490 healthcare workers′ mobile phone screens.

Resistance to methicillin (*n*, %)	*Coa* type/SCC*mec* type (*N*, %)	Antibiotic resistance profile^a^ (*N*; %)	Genetic resistance profile (no; %)	Biofilm status^b^ (*N*; %)
MSSA (22, 29.3)	X (8, 36.4)	PEN, GEN, ERY (2, 25)	*erm*(*A*) (2, 25), *erm*(*B*) (2, 25), *tet*(*M*) (4, 50), *ant* (4 ^′^)*-Ia* (2, 25)*, aac* (6 ^′^)*-Ie/aph* (2 ^″^) (2, 25)	S (5, 62.5), W (2, 25), M (1, 12.5)
		PEN, GEN, TET, ERY, CIP, SYN (2, 25)		
		PEN, GEN, TET, ERY (2, 25)		
		TET, CIP (2, 25)		
	III (6, 27.3)	PEN, GEN, TET, ERY, CLI, CIP (3, 50)	*erm*(*C*) (2, 33.3), *tet*(*M*) (2, 33.3), *ant* (4 ^′^)*-Ia* (3, 50)	S (2, 33.3), M (3, 50), W (1, 16.7),
		PEN, GEN, TER, ERY, CLI (1, 16.7)		
		GEN, RIF (2, 33.3)		
	IVb (5, 22.7)	TET, CIP (2, 40)	*tet*(*M*) (2, 40), *ant* (4 ^′^)*-Ia* (1, 20)	S (1, 20), M (2, 40), N (2, 40)
		PEN, CIP (2, 40)		
		GEN, RIF (1, 20)		
	VII (3; 13.6)	TET, CIP (2, 66.7)	*erm*(*A*) (1, 33.3), *tet*(*M*) (1, 33.3), *aph* (3 ^′^)*-IIIa* (1, 33.3)	N (2, 66.7), W (1, 33.3)
		CIP (1, 33.3)		
MRSA (53, 70.7)	II/III (10, 18.9), II/IV (7, 13.3)	PEN, TET, MUP, SXT, RIF (4, 23.6)	*mecA* (17, 100), *tet*(M) (15, 88.2), *mupA* (10, 58.8), *aac* (6 ^′^)*-Ie/aph* (2 ^″^) (6, 35.3), *aph* (3 ^′^)*-IIIa* (4, 23.5) *ant* (4 ^′^)*-Ia* (4, 23.5)*, erm*(*B*) (2, 11.8), *erm*(*A*) (4, 23.5), *msrB* (6, 35.3), *msrA* (2, 11.8)	S (11, 64.8), M (3, 17.6), W (3, 17.6)
		PEN, GEN, TET, RIF, MUP (4, 23.6)		
		PEN, GEN, TET, SXT, MUP (3, 17.6)		
		PEN, GEN, TET, ERY, SYN, NIT, SXT (3, 17.6)		
		PEN, ERY, CLI, CIP, RIF (3, 17.6)		
	I/IV (8, 15.1), I/II (6, 11.3)	PEN, GEN, TET, RIF, MUP (2, 14.3)	*mecC* (3, 17.6), *mecA* (11, 78.6), *erm*(*B*) (5, 35.7), *msrB* (3, 21.4), *tet*(*M*) (8, 57.1), *ant* (4 ^′^)*-Ia* (4, 28.6)*, erm*(*A*) (2, 14.3), *msrA* (4, 28.6)	S (9, 64.3), M (2, 14.3), W (3, 21.4)
		PEN, GEN, TET, SXT, MUP (2, 14.3)		
		PEN, GEN, TET, ERY, CIP, SYN (3, 21.4)		
		PEN, GEN, TET, ERY (6, 42.9)		
		GEN, RIF (1, 7.1)		
	Vb/V (8, 15.1), Vb/IV (4, 7.5)	PEN, GEN, TER, ERY, CLI (5, 41.7)	*mecA* (12, 100), *tet*(M) (3, 25), *aac* (6 ^′^)*-Ie/aph* (2 ^″^) (3, 25), *erm*(*A*) (3, 25), *msrB* (3, 25)	S (6, 50), M (3, 25), W (3, 25)
		PEN, GEN, TET, ERY, CLI, CIP (4, 33.3)		
		PEN, ERY, CLI, CIP, RIF (3, 25)		
	VII/IV (6, 11.3)	PEN, TET, CIP, NIT (2, 33.3)	*mecA* (6, 100), *ant* (4 ^′^)*-Ia* (2, 33.3), *tet*(M) (2, 33.3), *erm*(*B*) (2, 33.3), *msrA*(2, 33.3)	S (1, 16.7), M (4, 66.6), W (1, 16.7)
		PEN, CIP, SYN, NIT (2, 33.3)		
		TET, CIP (2, 33.4)		
	III/II (4, 7.5)	PEN, TET, CIP, NIT (2, 50)	*mecA* (4, 100), *tet*(M) (2, 50)	M (2, 50), W (2, 50)
		PEN, ERY, CLI, CIP, RIF (1, 25)		
		TET, CIP (1, 25)		

^a^CIP, ciprofloxacin; CLI, clindamycin; ERY, erythromycin; GEN, gentamicin; MUP, mupirocin; NIT, nitrofurantoin; PEN, penicillin; RIF, rifampicin; SXT, trimethoprim–sulfamethoxazole; SYN, quinupristin–dalfopristin; TET, tetracycline.

^b^S, strong; M, medium; W, weak.

## 4. Discussion

The global expansion of MDR staphylococci in both clinical environments and community settings has become a major public health concern. Personal electronic devices, particularly mobile phone screen surfaces, have increasingly been recognized as potential sites for microbial contamination. Contamination of these frequently touched surfaces may represent a possible factor associated with transmission risk in healthcare environments; however, direct transmission was not evaluated in the present study. [1, 4, 9]. In the present study, *S. aureus* contamination was detected in 15.3% of cell phones used by healthcare personnel. When interpreted in the context of previously published studies, this prevalence can be considered moderate. Higher mobile phone contamination rates have been reported in several geographical regions. For instance, studies conducted by Oluduro et al. in Africa [4], Hamdan‐Partida et al. in Latin America [5], and Goh et al. from Southeast Asia [10], have documented contamination levels of 31.6%, 53%, and 77.8%, respectively. In contrast, markedly lower prevalence rates have been described in countries such as Italy (4%) [11], Iran (3.3%) [12], and Japan (10.9%) [13]. These differences in *S. aureus* detection rates may reflect variations in infection control implementation, hand hygiene compliance, sampling approaches, and disinfection practices for personal electronic devices.

In the present study, MRSA constituted the majority of *S. aureus* isolates recovered from mobile phone screens, accounting for 70.7%, whereas MSSA represented 29.3% of isolates. This distribution differs from several previous investigations that reported a predominance of MSSA among mobile phone‐associated *S. aureus* isolates, suggesting considerable variability in MRSA contamination across different healthcare environments [4, 5]. The predominance of MRSA observed in this study may be explained by the frequent exposure of HCWs to colonized patients, contaminated clinical surfaces, and high‐risk hospital environments. Continuous handling of mobile phone screens during routine clinical activities may facilitate the transfer of resistant strains from healthcare settings to personal devices. These findings raise the possibility that contaminated mobile phone screen surfaces may serve as potential contact points for the persistence of MRSA within healthcare‐associated environments; however, direct transmission from phone screens was not assessed in the present study. The presence of resistant *S. aureus* isolates on HCWs′ mobile phone screen surfaces suggests that these devices may participate in microbial exchange networks within healthcare settings. However, the direction of transmission and the contribution of mobile phone screens to actual transmission events were not determined in this study.

CLI resistance among *S. aureus* isolates has been increasingly recognized as a clinically relevant therapeutic concern, as it can compromise the effectiveness of commonly used treatment regimens. In the present study, iMLS_B_ phenotype was detected in 24% of isolates. This prevalence differs from previously reported from Palestine (12.5%) [14] and Iran (7.8%) [8], indicating possible geographic and epidemiological variability in the distribution of MLS_B_ resistance mechanisms.

Mupirocin remains an important topical antimicrobial agent for limiting colonization and transmission of *S. aureus* in both healthcare facilities and community environments, particularly in strategies targeting nasal carriage and prevention of invasive infections [15]. However, the progressive emergence of mupirocin resistance, especially among MRSA strains, has become an increasing global concern that may compromise decolonization protocols. In the current study, mupirocin‐resistant *S. aureus* isolates accounted for 20% of all isolates, with a prevalence of 13.3% for *mupA* carrying HLMUPR isolates. The prevalence of mupirocin resistance reported worldwide varies considerably. For example, studies from North America (12.1%), Asia (Iran 39.6%; Turkey 45.5%; China 7%), and parts of Africa (27.8%) have documented a wide range of resistance frequencies, indicating substantial geographical variability [15]. The underlying drivers of increasing mupirocin resistance remain incompletely understood; however, multiple contributing factors have been proposed. These may include inappropriate or excessive topical antibiotic use, insufficient antimicrobial stewardship oversight, and limited regulation of antibiotic prescribing practices. Collectively, these factors may facilitate selective pressure that promotes the persistence and spread of resistant *S. aureus* lineages. These findings emphasize the importance of continuous surveillance of mupirocin susceptibility, particularly in healthcare‐associated MRSA populations, to preserve the effectiveness of decolonization strategies and prevent further dissemination of resistant strains.

The *mecC* gene has been identified in *S. aureus* from both humans and livestock, and its prevalence appears to be increasing [16]. In this study, 2.8% of clinical isolates carried *mecC*, all confirmed as MRSA. In Iran, *mecC* frequency has risen from 0.6% to 0.9% over the past two decades [17]. A meta‐analysis by Diaz et al. reported very low *mecC* prevalence in human isolates (0.04%) but higher levels in animal‐derived strains (0.10%) [18]. Global data show wide variability, ranging from 96.8% in Pakistan to 0.45% in the United Kingdom, with intermediate rates in other countries [18, 19]. The rising prevalence emphasizes the need for vigilance in monitoring *mecC* dissemination in both human and animal populations. The detection of *mecC*‐positive isolates in the present study deserves attention. Unlike *mecA*, which represents the predominant genetic determinant responsible for methicillin resistance in *S. aureus*, *mecC* is less frequently reported and has been mainly associated with specific ecological niches, including livestock‐associated reservoirs. Therefore, identification of *mecC*‐positive isolates among *S. aureus* recovered from mobile phone surfaces highlights the importance of continued molecular surveillance of methicillin resistance determinants. However, the present study was not designed to investigate the origin, transmission pathway, or epidemiological dissemination of *mecC*; therefore, further studies involving broader sampling and molecular epidemiological approaches are required. [16].

In the current investigation, the iMLS_B_ resistance phenotype was most frequently associated with the *erm*(B) determinant (38.9%), followed by *msr*(A) (27.8%), *erm*(A) (16.7%), *msr*(B) (11.1%), and *erm*(C) (5.5%). These data further support the well‐established contribution of ribosomal methylase and efflux pump mediated mechanisms to macrolide–lincosamide resistance in *S. aureus*. The relatively lower detection frequency of *erm*(C) in the present study remains within the range previously described. For example, Fasihi et al. reported detection rates of 20.5% and 11% for *erm*(C) and *erm*(A), respectively [20]. Moreover, experimental evidence provided by Lim et al. demonstrated that among ERY resistance–associated *erm* genes, *erm*(C) exhibited horizontal transfer capability in transformation assays, highlighting its potential epidemiological relevance.

The prevalence of *msr*(A) observed in our isolates (27.8%) is broadly consistent with the findings of Sedaghat et al., who reported a prevalence of 43.6% [21], indicating sustained circulation of efflux‐mediated resistance determinants in clinical *S. aureus* populations. Similarly, the detection frequency of *erm*(B) (38.9%) in the present study aligns with reports from geographically distinct regions, including a study from Texas documenting a prevalence of 46.3% [22]. Taken together, these findings emphasize the multifactorial genetic basis of MLS_B_ resistance and underscore the necessity for continuous molecular surveillance to monitor the evolving epidemiology of antimicrobial resistance in *S. aureus*.

Our analysis indicates that GEN resistance among the examined *S. aureus* isolates (46/75) was predominantly mediated by the *ant*(4 ^′^)‐Ia gene, identified in 34.8% (16/46) of resistant strains, whereas *aac*(6 ^′^)‐*Ie/aph*(2 ^″^) (23.9%; 11/46) and *aph*(3 ^′^)‐*IIIa* (10.9%; 5/46) contributed to a lesser extent. The marked heterogeneity in the distribution of *ant*(4 ^′^)‐Ia across studies highlights the dynamic nature of aminoglycoside resistance epidemiology. For example, Ida et al. reported an exceptionally high prevalence of this determinant (84.5%) [23], a finding that likely reflects intense selective pressure and efficient horizontal gene transfer within dominant MRSA clones. In contrast, the more moderate prevalence observed in the present study parallels reports from Ardic et al. [24] and Perumal et al. (9%) [25], suggesting regional variability rather than uniform dissemination of this resistance mechanism.

Notably, the relatively low occurrence of *aac*(*6*  ^′^)*-Ie/aph*(*2*  ^″^) in our isolates (14.7%) contrasts sharply with the substantially higher prevalence documented by Ardic et al. (60.5%) [24], underscoring the influence of local antimicrobial prescribing practices on the genetic landscape of resistance. Similarly, the infrequent detection of *aph*(*3*  ^′^)*-IIIa* (6.7%) is consistent with earlier report from Ardic et al. (8%) [24], collectively indicating that this determinant plays a limited role in GEN resistance among contemporary *S. aureus* populations. Taken together, these findings demonstrate the presence of aminoglycoside resistance phenotypes and associated resistance determinants among *S. aureus* isolates recovered from HCWs′ mobile phone screen surfaces. The detection of these resistance markers highlights the importance of continued surveillance of resistant *S. aureus* strains in healthcare‐associated environments. The convergence of multiple resistance determinants, even at moderate prevalence levels, suggests that continued reliance on aminoglycosides may be increasingly ineffective, particularly in settings with sustained selective pressure. These findings emphasize the importance of antimicrobial resistance surveillance and monitoring of resistance determinants among *S. aureus* isolates recovered from healthcare‐associated environments.

One of the key challenges in reducing microbial contamination on mobile phone screen surfaces is the ability of bacteria to persist in biofilm‐associated forms on frequently touched device surfaces. In the present study, an overwhelming majority (94.7%) of *S. aureus* isolates demonstrated the capacity to produce biofilms, with 49.3% classified as strong, 28.2% as moderate, and 22.5% as weak producers. Prior findings from Mexico indicated universal biofilm‐forming capability among the tested *S. aureus* isolates [5]. In addition, they observed that nearly half of the isolates demonstrated weak biofilm formation, whereas 23.4% and 29.8% displayed moderate and strong biofilm‐producing ability, respectively [5]. These findings highlight the widespread potential of *S. aureus* to establish biofilms on personal devices, supporting the notion that such structures contribute to microbial persistence in both community and healthcare settings. The frequent detection of MDR isolates among biofilm‐producing strains highlights the coexistence of these two clinically relevant characteristics. However, because statistical association analysis was not performed, the present findings should be interpreted as descriptive rather than demonstrating a direct relationship between biofilm formation intensity and antimicrobial resistance.

To elucidate the molecular basis underlying biofilm development, the presence of several key adhesion‐related genes was assessed. Among the isolates, the most frequently identified biofilm‐related gene combinations were *clfA, clfB, fnbA, fnbB; clfA, clfB, fnbA, fnbB, cna;* and *clfA, clfB, fnbA, fnbB, cna, ebp,* occurring at rates of 22.5%, 19.7%, and 19.7%, respectively, reflecting their critical involvement in initial attachment and subsequent biofilm maturation. The present study highlights the critical role of multiple adhesion and biofilm‐associated genes in *S. aureus* isolates from mobile phones. In line with our observations, previous report by Noumi et al. identified *fnbA*, *cna*, and *icaA* as frequently occurring determinants contributing to biofilm formation [26], whereas other investigations have emphasized the prevalence of *clfB*, *sdrC*, and *icaD* in device‐associated isolates [5]. Moreover, in a study conducted in Iran, genes such as *icaA*, *fnbA*, and *clfA* have been consistently detected in clinical *S. aureus* strains recovered from urinary tract infections, underscoring their widespread involvement in biofilm development. The distribution of these genes suggests that multiple adhesion mechanisms operate synergistically to stabilize biofilm architecture, thereby facilitating persistence on mobile phone surfaces.

In this study, the majority of *S. aureus* isolates carried the SCC*mec* Type IV [47.1% (25/53)]. Previous investigation conducted by Prakash et al. in Iran has reported a high prevalence of SCC*mec* Type IV among clinical isolates, reflecting the contribution of community‐associated strains in the region [27]. Interestingly, strains with strong biofilm‐forming ability were frequently associated with SCC*mec* Type IV, suggesting a potential link between this genetic element and enhanced biofilm production. These observations underscore the interplay between methicillin resistance determinants and biofilm‐forming capacity in shaping the persistence and epidemiology of *S. aureus* in healthcare settings.

*Coa* typing of the 75 *S. aureus* isolates identified seven distinct *coa* types, designated as II, I, Vb, III, VII, X, and IVb. The relative prevalence of these types was 22.7% (17/75), 18.7% (14/75), 16% (12/75), 13.3% (10/75), 12% (9/75), 10.7% (8/75), and 6.6% (5/75), respectively, indicating diversity among the investigated *coa* types and molecular markers. Further high‐resolution approaches, such as *spa* typing, MLST, or whole‐genome sequencing, are required to determine clonal relationships and population‐level genetic diversity. These findings are consistent with previous reports; for instance, Afrogh et al. identified nine different *coa* patterns among nasal swabs from healthcare personnel and clinical samples in Iran, demonstrating comparable genetic diversity [28]. Similarly, studies from Thailand and Egypt have documented a wide distribution of *coa* types, corroborating the heterogeneity observed in our isolates [29, 30].

In our analysis, *coa* Types II, I, and Vb were the most prevalent, accounting for 22.7%, 18.7%, and 16% of the isolates, respectively. In agreement, Hirose et al., using the same primer set, detected seven distinct *coa* types, reporting a predominance of Type II among MRSA isolates (91.9%), followed by Types VII (3.9%), I (1.7%), IVa (1.1%), III and X (0.6% each), and VI (0.2%), with two MRSA isolates remaining untypeable [7]. The observed differences in *coa* type distribution across studies likely reflect geographical variation and local epidemiological factors influencing the circulation of specific *S. aureus* lineages.

The recovery of MRSA from cell phones used by HCWs represents a critical infection control concern, as these devices may function as persistent reservoirs facilitating the circulation of clinically important *S. aureus* clones. Notably, the predominance of *coa* Types II and I among *S. aureus* isolated cell phones may indicate the epidemiological success of these lineages and suggests their potential for broader dissemination within Iran in the absence of targeted surveillance and control measures [31, 32].

Considering the detection of MDR *S. aureus* isolates and multiple resistance‐associated determinants on mobile phone screen surfaces, several precautionary measures may be considered to reduce microbial contamination of frequently handled personal devices in healthcare settings. These measures may include improving awareness regarding hand hygiene before and after mobile phone use, regular cleaning and disinfection of device surfaces using appropriate methods compatible with electronic equipment, and establishing institutional guidelines for mobile phone handling in high‐risk healthcare environments. However, the effectiveness of specific decontamination strategies was not evaluated in the present study.

This study has several limitations. First, the cross‐sectional design and single‐time sampling of mobile phone screen surfaces do not allow assessment of temporal relationships or direct transmission pathways. Second, samples from HCWs′ hands, patients, and surrounding hospital environments were not collected simultaneously; therefore, the direction of bacterial transfer and the causal contribution of mobile phone contamination to pathogen dissemination could not be established. Third, the association between biofilm production intensity and antimicrobial resistance was not statistically evaluated. Therefore, further studies with appropriate analytical approaches are required to determine whether biofilm formation intensity is significantly associated with resistance phenotypes. Fourth, although *coa* and SCC*mec* typing provided useful information regarding variation among selected molecular markers, higher‐resolution approaches such as *spa* typing, MLST, or whole‐genome sequencing would be required to define clonal relationships and population‐level genetic diversity among these isolates. Fifth, the absence of paired sampling from device owners, HCWs, patients, and surrounding environments prevented assessment of bacterial transfer pathways and direct verification of dissemination. Another limitation of this study is that hospital‐specific information and comparative analyses between individual healthcare centers were not performed. Finally, although PCR‐based detection provided valuable information regarding the presence of antimicrobial resistance‐ and adhesion‐associated genetic determinants, the detection of these genes does not necessarily indicate their expression or functional activity. Since gene expression analyses were not performed in this study, the contribution of these genetic determinants to the observed phenotypic characteristics requires further investigation using functional and transcriptomic approaches.

## 5. Conclusion

In conclusion, this study demonstrated the presence of *S. aureus* on the mobile phone screen surfaces of HCWs, with a high proportion of methicillin‐resistant and MDR isolates. A considerable proportion of the isolates exhibited biofilm‐forming ability and carried resistance‐associated determinants, including *mecA*, *tetM*, and aminoglycoside‐ and macrolide‐resistance genes. Molecular characterization revealed diversity in *coa* types and SCC*mec* profiles, alongside a high prevalence of adhesion‐associated genes. These findings characterize the antimicrobial resistance and virulence‐associated features of *S. aureus* isolates recovered from HCWs′ mobile phone screens and provide baseline data for further epidemiological research. However, given the cross‐sectional design and the absence of paired samples from HCWs or patients, this study cannot establish whether contaminated mobile phone screens contribute to pathogen transmission. Longitudinal studies incorporating paired sampling and molecular epidemiological approaches are needed to clarify the potential role of mobile phones in the transmission of *S. aureus*.

## Author Contributions

M.G. conceived and designed the experiments, performed the experiments, analyzed and interpreted the data, and wrote the paper. A.N. performed the experiments; analyzed and interpreted the data, materials, and analysis tools; and wrote the paper.

## Funding

This study was supported by the Shahid Beheshti University of Medical Sciences (10.13039/501100005851; 43020470).

## Ethics Statement

This study was approved by the Ethics Committee of the Shahid Beheshti University of Medical Sciences in Tehran, Iran (IR.SBMU.MSP.REC.1405.265). Written informed consent was obtained from all participants after they were fully informed about the study′s objectives and methods.

## Conflicts of Interest

The authors declare no conflicts of interest.

## Data Availability

The data that support the findings of this study are available from the corresponding author upon reasonable request.
